# DNA methylation drives a new path in gastric cancer early detection: Current impact and prospects

**DOI:** 10.1016/j.gendis.2023.02.038

**Published:** 2023-03-30

**Authors:** Xinhui Wang, Yaqi Dong, Hong Zhang, Yinghui Zhao, Tianshu Miao, Ghazal Mohseni, Lutao Du, Chuanxin Wang

**Affiliations:** a Department of Clinical Laboratory, The Second Hospital of Shandong University, Jinan, Shandong 250033, China; b Department of Clinical Laboratory, Fuling Hospital, Chongqing University, Chongqing 402774, China; c Suzhou Research Institute of Shandong University, Suzhou, Jiangsu 215123, China; d Department of Biochemistry and Molecular Biology, Shandong University School of Basic Medical Sciences, Jinan, Shandong 250012, China; e Shandong Engineering & Technology Research Center for Tumor Marker Detection, Jinan, Shandong 250033, China; f Shandong Provincial Clinical Medicine Research Center for Clinical Laboratory, Jinan, Shandong 250033, China

**Keywords:** Biomarkers, DNA methylation, Early detection, Gastric cancer, Translation

## Abstract

Gastric cancer (GC) is one of the most common and deadly cancers worldwide. Early detection offers the best chance for curative treatment and reducing its mortality. However, the optimal population-based early screening for GC remains unmet. Aberrant DNA methylation occurs in the early stage of GC, exhibiting cancer-specific genetic and epigenetic changes, and can be detected in the media such as blood, gastric juice, and feces, constituting a valuable biomarker for cancer early detection. Furthermore, DNA methylation is a stable epigenetic alteration, and many innovative methods have been developed to quantify it rapidly and accurately. Nonetheless, large-scale clinical validation of DNA methylation serving as tumor biomarkers is still lacking, precluding their implementation in clinical practice. In conclusion, after a critical analysis of the recent existing literature, we summarized the evolving roles of DNA methylation during GC occurrence, expounded the newly discovered noninvasive DNA methylation biomarkers for early detection of GC, and discussed its challenges and prospects in clinical applications.

## Background

Gastric cancer (GC) ranks the fifth most common cancer worldwide, with an estimated 11,090 deaths in the United States in 2022.^1^ Even if significant advances have been made in screening and treatment strategies, this malignancy remains one of the most devastating diseases, with a median overall survival of only ∼14.2 months in advanced patients.^2^ Before GC onsets, it slowly undergoes a series of multistep intermediate stages in the following order: superficial gastritis, atrophic gastritis, intestinal metaplasia, gastric epithelial dysplasia, and eventually carcinogenesis, generally following years or decades.^3^ If this malignancy can be detected in a precancerous or early stage, the 5-year survival rate can be greatly improved.^4^ The tumorigenesis of GC involves multiple factors closely related to epigenetic regulation, and comprehensive research on the pathogenesis at the epigenetic molecule level offers a novel opportunity for the early detection of GC.^5^ In the era of molecular diagnostics, DNA methylation, as the prominent epigenetic process, is essential for solving clinical problems such as cancer screening, detection, and risk prediction.

DNA methylation, as early as 1948, was defined by scientists as a 5-methylcytosine (5 mC) formation process from the transfer of methyl donors of s-adenosylmethionine (SAM) on cytosine, which was catalyzed by DNA methyltransferases (DNMTs).^6^ 5 mC is linked to guanosine via a DNA phosphate group to form cytosine-guanine dinucleotide (CpG) sites, and specific regions of CpG enrichment are called CpG islands (CGIs).^7^ DNA methylation can alter gene expression without changing the gene sequence, leading to changes in DNA conformation, chromatin structure, and DNA stability, which control gene expression.^8^^,^^9^ Typically, most CGI-containing gene promoter regions are unmethylated. However, abnormal CGI-hypermethylation of tumor suppressor genes and the suppression of subsequent gene expression are critical initial events in malignancy.^10^^,^^11^ Since the introduction of genomic DNA methylation profiling, several studies have proposed that epigenetics-based DNA methylation detection plays a crucial role in early risk screening of GC.^12^^,^^13^ In the present review, we described the role and latest progress of DNA methylation as an epigenetic hallmark in the pathogenesis and early screening of GC. In addition, we also discussed the current limitations and prospects of DNA methylation research. Taken together, our present review would help translate these findings on DNA methylation in GC areas to clinical practice.

## DNA methylation and GC

**Figure 1:**
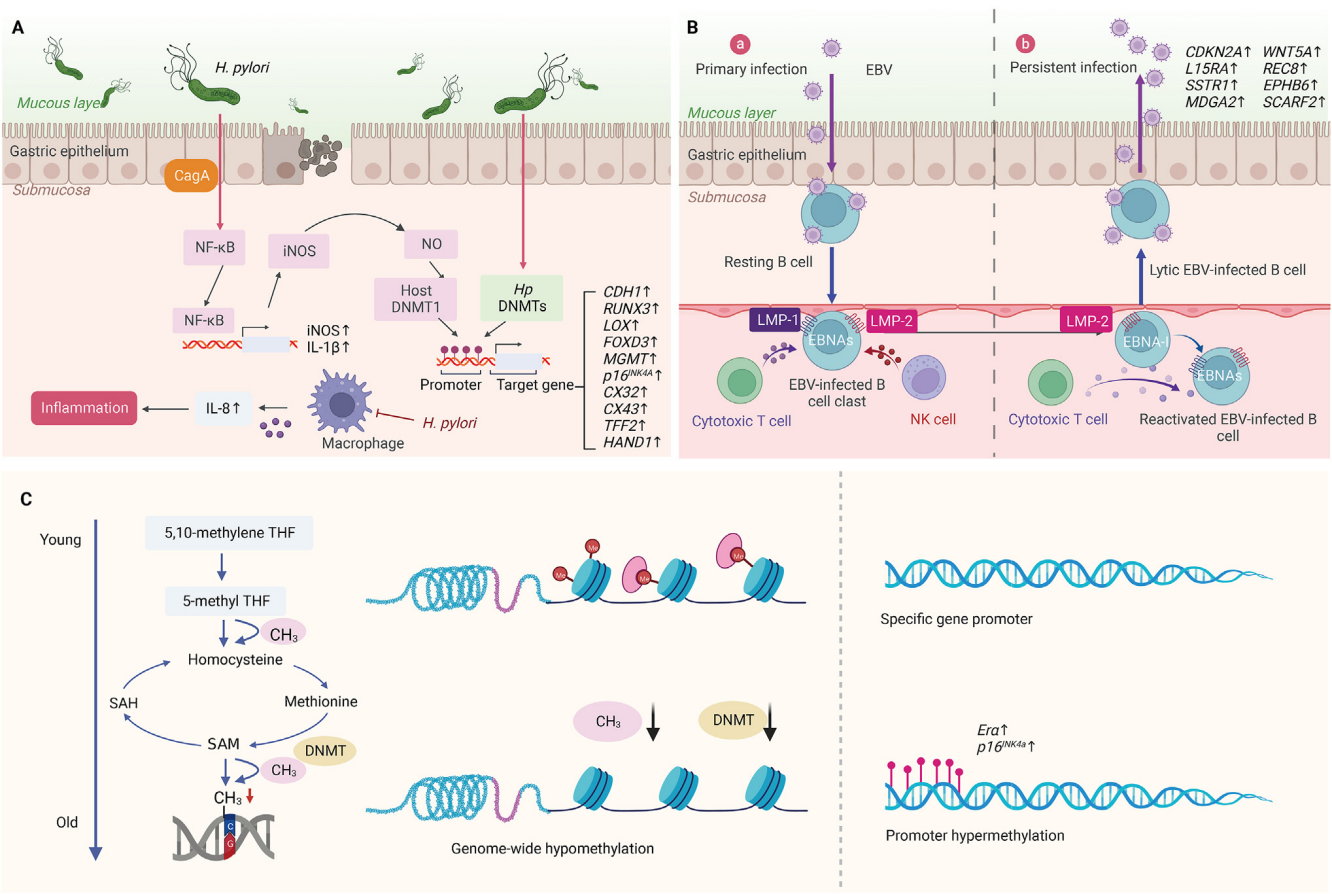
Schematic representation of DNA methylation role during GC. **(A)***H. pylori* enters gastric epithelial cells through CagA, activates NF-κB to trigger inflammation, and increases the expressions of cytokines (IL-1β, IL-8) and NO. NO-activated host DNMT-1 and *H. pylori*'s DNMTs together lead to abnormal DNA methylation of GC-related genes. **(B)** EBV enters gastric epithelial cells through contact with EBV-infected B lymphocytes, inducing the accumulation of aberrant DNA methylation in cells via their membrane proteins LMP-2A and LMP-1. **(C)** The global DNA hypomethylation and hypermethylation in CGIs of some specific gene promoters caused by aging. The aging-induced deceleration of one-carbon cycle metabolism results in a decrease in methyl donors and reduces the activities of DNMTs. CagA, cytotoxin-associated gene A; DNMTs, DNA methyltransferases; EBV, Epstein-Barr virus; *H. pylori,Helicobacter pylori*; IL-1β, interleukin-1β; IL-8, interleukin-8; iNOS, induced nitric oxide synthase; LMP, latent membrane protein; NF-κB, nuclear factor kappa-B; NO, nitric oxide; PMN, polymorphonuclear neutrophils; SAM, s-adenosylmethionine.

During normal cellular physiological processes, DNA repetitive elements (REs), making up about 50% of the human genome, require DNA methylation to suppress mobility and maintain genome stability.^14^ The alterations in DNA methylation deregulate the genome, as is evident from the extensive changes in DNA methylation patterns observed in the human cancer genome, including DNA hypomethylation of most REs and hypermethylation of numerous CGIs.^15^^,^^16^ In addition, accumulating evidence has shown that pathogens and aging are associated with the build-up of DNA methylation aberrations in gastric tissue (Fig. 1). Notably, the accumulation and modification of DNA methylation patterns have been linked to risk factors for GC.^17^^,^^18^ Therefore, understanding the potential mechanisms of DNA methylation during GC is of research value and clinical significance.

### DNA methylation is the bridge between Helicobacter pylori and GC

*Helicobacter pylori* (*H. pylori*) is a spiral-shaped Gram-negative microaerobic bacterium that is acid-resistant compared with other bacteria and, therefore, can survive inside the stomach.^19^ As one of the most prevalent infectious agents worldwide, *H. pylori* infects more than the global average population.^20^ Since *H. pylori* can trigger the Correa's cascade and play a causative etiological role in gastroduodenal diseases such as gastritis, digestive tract ulcer, and lymphoproliferative gastric lymphoma, it is generally regarded as a significant causal factor of GC development.^21^ Epidemiological statistics show that nearly 1 million new GC cases and 738,000 GC-related deaths occur worldwide annually, with *H. pylori* responsible for an estimated 89%.^22^^,^^23^ A previous study has shown abnormal DNA hypermethylation in the promoter region of specific genes due to *H. pylori* infection by comparing DNA methylation levels in gastric mucosal specimens collected via gastroscopy from 35 patients (with the presence of *H. pylori* infection) and 11 healthy volunteers, and the hypermethylation status persists even after eradication of *H. pylori*.^24^ However, this persistent hypermethylation can be suppressed by immunosuppressive therapy. Eradicating *H. pylori* in gerbils does not inhibit DNA hypermethylation in gastric epithelial cells, while cyclosporin-A does.^25^ Another study on gerbils also supports this result, showing that a demethylating agent (5-aza-2′-deoxycytidine) effectively prevents GC caused by *H. pylori* infection through DNA demethylation.^26^

*H. pylori*-induced aberrant DNA methylation plays a triggering role in the occurrence of GC; DNA methylation is the bridge between *H. pylori* and GC. The oncogenicity of *H. pylori* depends on cytotoxin-associated gene A (CagA), which induces abnormal methylation of the gene promoter, leading to carcinogenesis in normal gastric cells.^27^ Ectopic CagA directly regulates the expression of oncogene *RAS* by promoting the hypermethylation-induced silencing of microRNA let-7.^28^ In addition, clinical research has also demonstrated that *H. pylori* can raise the risk of GC by inducing DNA methylation in chronic gastritis. For example, *H. pylori*-infected gastric epithelial cells promote nitric oxide production, leading to hypermethylation of the *RUNX3* gene in epithelial cells.^29^ Moreover, *H. pylori* can up-regulate cytokines IL-1β, TNF, and IL-8, which may be a common factor in increasing the risk of GC by inducing abnormal DNA methylation.^30^ Previous studies believe that it is mainly due to the formulation and accumulation of aberrant DNA methylation, leading to the development of inflammation and spread of *H. pylori* and then promoting GC occurrence.

### The unique role of Epstein-Barr virus (EBV)-induced DNA methylation in GC

EBV is a genus of herpes viruses for which humans are the primary host, and about 90% or more of adults have antibodies to the virus.^31^ Burke et al first detected EBV DNA in paraffin-embedded blocks of undifferentiated lymphoepithelial GC by PCR in 1990.^32^ EBV-associated gastric carcinoma (EBVaGC) frequency ranges from 1.3% to 30.9% in different regions, with an average of 10% worldwide.^33^

Although the specific pathogenesis of EBVaGC is unknown, EBV can induce hypermethylation in both host and viral genomes, which is consistent with the fact that the primary pathogenesis of EBVaGC is hypermethylation in the specific promoter regions of individual genes.^34^^,^^35^ Kaneda et al have found that EBV infection in hypermethylated GC cell lines results in their *de novo* methylation within approximately 18 weeks, transforming them into EBV-positive methylated cell lines and suppressing the expressions of multiple oncogenes.^36^ Vo et al analyzed 107 samples using methylation-specific PCR (MSP), including 96 EBV-negative gastric carcinomas (EBVnGC) and 11 EBVaGC samples and compared them with EBVnGC, and found high methylation of *CDKN2A* promoter in EBVaGC.^37^ Zhao et al demonstrated EBV-driven hypermethylation by a human gastric epithelial cancer cell line AGS. They found that the expressions of DNMT-3b and most differentially hypermethylated genes (83.2%, 886 of 1065 genes) were significantly increased in EBV-positive AGS cells compared with uninfected cells (fold change 2.43∼65.2), particularly, among which, six GC-related genes, *SCARF2*, *REC8*, *IL1*5RA, *EPHB6*, *SSTR1*, and *MDGA2*, are identified by bisulfite genome sequencing.^38^ The above studies indicate that EBV plays a unique role during the occurrence and development of GC and EBVaGC should be regarded as a distinct entity in the GC population.

### Aging leads to DNA methylation alterations associated with GC

The incidence of GC rises progressively with age, while gastric adenocarcinoma rarely occurs in patients under 30 years.^39^ Aging is a highly complex biochemical process that involves molecular-level genomic instability, including changes in DNA methylation patterns that are common signs of aging and cancer. Therefore, age-dependent DNA methylation may explain the relationship between aging and increased cancer incidence.^40^ Genome-wide single nucleotide resolution analysis shows that centenarians have hypermethylation in CGIs of the promoter regions and global DNA hypomethylation compared with neonates, which is similar to the characteristics of cancer epigenome.^41^ During *in vitro* passaging of mouse diploid fibroblasts, the content of genomic 5-mC is significantly decreased with an increasing number of culture generations. In contrast, the incidence of methylation is relatively more stable in immortalized mouse cell lines.^42^ For most vertebrates, age-related DNA methylation follows specific patterns, while the methylation level of the promoter regions in senescent cells is inconsistent for some specific individual genes.^43^ In addition to the overall reduction of genome methylation, some promoter regions of specific genes are prone to hypermethylation, such as *ERα* and *p16*^*INK4a*^, which have been confirmed to be tumor suppressor genes in GC.^44^, ^45^, ^46^, ^47^, ^48^

The underlying molecular links of aging leading to GC-associated DNA methylation alterations remain elusive, and several studies have attempted to answer this question. First, the activity levels of DNMT-1 in the body are essential endogenous regulatory factors of DNA methylation, which play a crucial role in maintaining the hypermethylated state of heterochromatin DNA.^49^ Connelly et al have reported that from birth to aging, the activity and expression levels of DNMT-1 are reduced, leading to passive demethylation, and the DNA methylation of the genome is gradually depleted.^50^ In another study of Wistar rats aged 3–20 months, Elsner et al have found that hypermethylation of H3-K9 and DNMTs may be associated with the brain aging process.^51^ Another explanation is that the intake and availability of folic acid, one of the methyl donors, is decreased with age, which may also lead to hypomethylation of the whole genome.^52^ From another perspective, the blockade of the one-carbon metabolic pathway involving folic acid may cause the accumulation of homocysteine in the blood and increase the cellular S-adenosylhomocysteine, further inhibiting the DNMT activity and resulting in a persistent hypomethylation state of the genome.^53^

Horvath et al first proposed a breakthrough in 2013, which is to construct an age predictor based on DNA methylation in multiple human tissues to capture the degree of human biological aging.^54^ Extensive progress has recently been made in developing biomarkers to measure “biological age” within this framework. By bisulfite pyrosequencing of healthy samples, Weidner et al have constructed an epigenetic aging signature using plasma samples, including three age-related CpGs located in the genes *PDE4C*, *ASPA*, and *ITGA2B* that facilitate age predictions.^55^ Considering the tissue specificity of methylation levels, Stubbs et al have constructed a multi-tissue DNA methylation-based epigenetic clock as a chronological age predictor in mice.^56^ Hong et al employed Horvath's clock model in 591 GC tissue samples and 115 normal gastric tissues and found that the model accurately captures age in normal tissues but not in cancer tissues.^57^ Compared with normal tissues, the predicted age of DNA methylation in GC tissue is lower than its chronological age. This shift has excellent potential for early diagnosis and application in GC patients.

## DNA methylation ignites a revolution in the early detection of GC

As GC is an insidious disease, a significant proportion of patients only have unexplained weight loss and indigestion in the early stage, which is not enough to attract the attention of patients, resulting in a delayed diagnosis of GC.^58^ The 5-year survival rate for GC is only 32.4% in the United States, mainly because approximately 60% of cases are diagnosed at an advanced stage.^59^ However, several countries with a high incidence of GC have established screening programs to improve the early detection of GC and overall survival rates. For example, in Japan, the early diagnosis of GC reaches 50%, and the 5-year survival rate attains 95% if cancers confined to the stomach wall lining are diagnosed early.^60^^,^^61^ The East-West difference for this disease, such as the higher incidence but lower mortality rates in Japan and South Korea, is mainly attributed to the widespread use of endoscopy.^62^^,^^63^ However, as recommended in South Korea and Japan, endoscopy is challenging to accept in other countries due to poor compliance, and the approach is not affordable in countries with low socio-economic levels.^64^^,^^65^ Therefore, non-invasive and affordable biomarkers that can accurately identify GC at an early stage or precancerous lesions stages are of most interest in countries with low disease incidence, such as accidents, especially if such biomarkers can detect the disease during the window period between disease onset and symptom development.^66^

Biomarkers are objective indicators of tumor development and can monitor the response of tumors to treatment.^67^ In clinical practice, carbohydrate antigen (CA) 19-9 and carcinoembryonic antigen are the most common conventional biomarkers used before and after GC surgery.^68^^,^^69^ In addition, it has been reported that CA125 and CA72-4 can be elevated in advanced GC.^70^ Although the above biomarkers can be used for auxiliary diagnosis treatment response evaluation of GC and monitoring for recurrence after successful treatment, the positive rate of these biomarkers in an early stage is less than 10%, and their sensitivity and specificity in detecting early GC are relatively low. Therefore, they are not recommended as a primary indicator for early detection of GC.^71^

**Figure 2:**
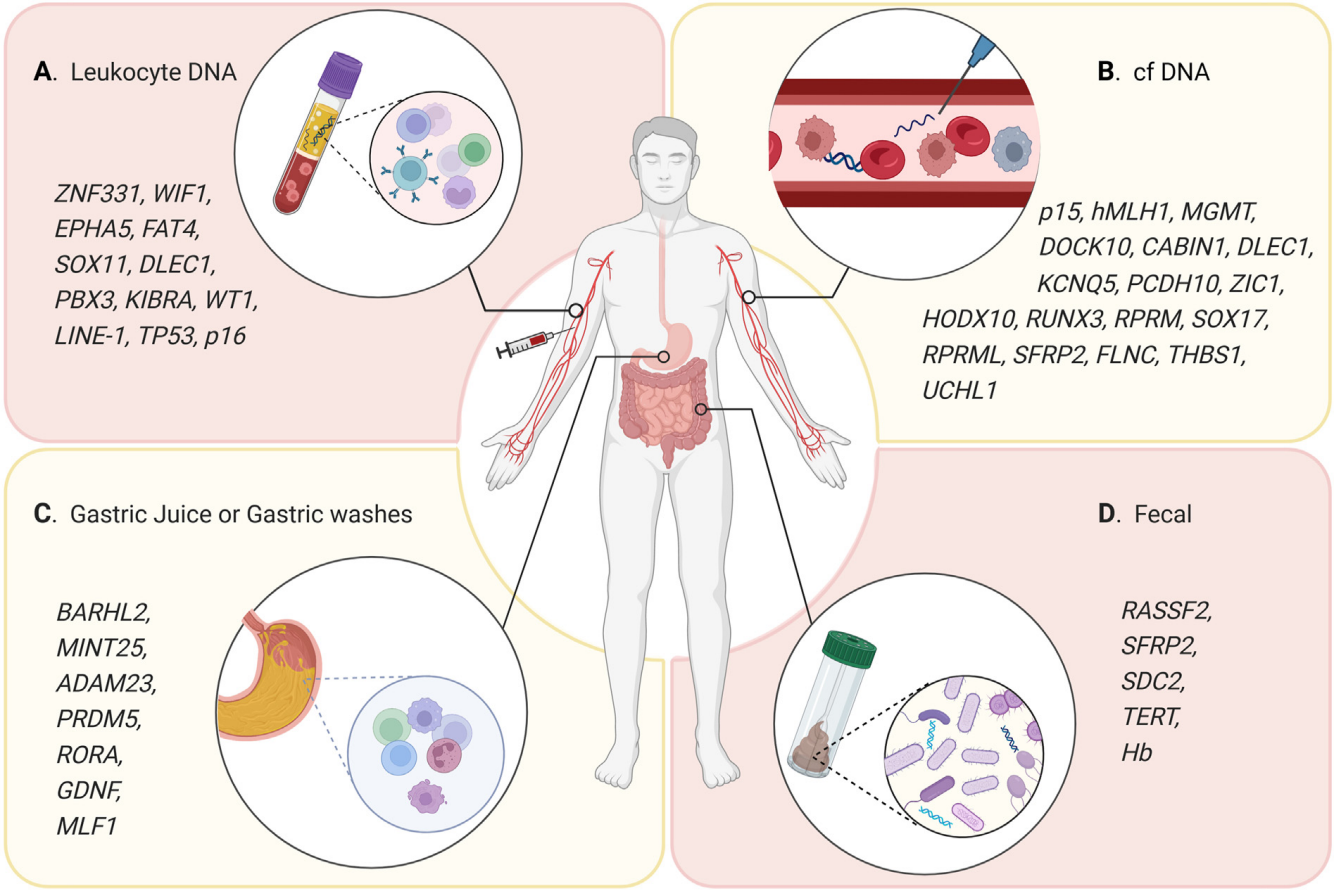
The application of novel DNA methylation biomarker candidates for the early detection of GC from different sample sources: **(A)** leukocytes DNA, **(B)** cfDNA, **(C)** gastric juice or gastric washes, and **(D)** feces.

The latest advances in sequencing technology lead to a deeper exploration of the complex genomic landscape of GC, and aberrant DNA methylation occurs in the early stages of GC, even before the occurrence of cancer.^17^^,^^72^ In theory, early detection of abnormal methylation site profiles associated with GC can play a role in early detection. In practice, by sequencing and analyzing cancer-related methylation sites, scientists have attained DNA methylation candidates for early detection of GC^73^^,^^74^ (Table 1). However, most current studies on aberrant DNA methylation have focused on assessing the difference in GC tissue methylation levels.^75^, ^76^, ^77^, ^78^ In this case, surgically obtained gastric tissue is required for testing, significantly limiting the clinical use of DNA methylation markers. Therefore, the researchers are seeking a simpler, less invasive way to collect samples and detect DNA methylation. Since methylation-related changes in cancer patients are systematic, accumulating research has shown that aberrant methylation can also be detected in peripheral blood, gastric juice, and feces, making up a valuable biomarker for early GC screening (Fig. 2).

**Table 1:** Aberrant DNA methylation as a biomarker in body fluids and feces of GC patients.

Methylated sites	Cohort size		Sensitivity (%)	Specificity (%)	Method	Reference
	Controls	GCs				
***Cell-free DNA samples***						
*p15/hMLH1*	22	20	65	72	MSP	^79^
*MGMT/p15/hMLH1*	22	20	75	54	MSP	
*DOCK10/CABIN1/KCNQ5*	82	89	64	93	MCTA-Seq	^74^
*PCDH10*	202	101	94.1	97.03	MSP	^80^
*ZIC1*	34	131	69.5	69.2	MSP	^81^
*HODX10*	34	131	48.1	80	MSP	
*RUNX3*	34	131	42.7	79.2	MSP	
*ZIC1/HODX10*	34	131	82.4	58.3	MSP	
*ZIC1/RUNX3*	34	131	83.2	57.5	MSP	
*HODX10/RUNX3*	34	131	72.5	65	MSP	
*ZIC1/HODX10/RUNX3*	34	131	91.6	50	MSP	
*RPRM*	88	96	47	93	MSP	^82^
*RUNX3*	88	96	59	95	MSP	
*RPRM/RUNX3*	88	96	82	89	MSP	
*RUNX3*	120	202	94.1	100	MSP	^83^
*SOX17*	20	73	58.9	100	MSP	^84^
*RPRML*	25	25	56	88	MethyLight	^85^
*SFRP2*	50	92	60.9	86	Q-PCR	^86^
*FLNC*	40	82	67.1	93	Q-MSP	^87^
*THBS1*	40	82	63.4	94.2	Q-MSP	
*UCHL1*	40	82	56.1	89.5	Q-MSP	
*DLEC1*	40	82	80.5	93	Q-MSP	
***Fecal samples***						
*TERT*	62	35	52.2	90	Pyrosequencing	^88^
*SDC2*	90	66	40.9	93.3	PCR	^89^
*TERT*	90	66	36.4	90	PCR	
*RASSF2*	90	66	31.8	93.3	PCR	
*SFRP2*	90	66	22.7	90	PCR	
*Hb*	90	66	27.3	90	PCR	
*SDC2/TERT/Hb*	90	66	66.7	78.9	PCR	
*RASSF2/SFRP2*	101	21	57.1	89.4	Hi-SA	^90^
*WIF1/SDC2/TFPI2/NDRG4*	107	35	67.5	97.81	ColoCaller	^91^
***Gastric juice or washes***						
*BARHL2*	32	128	90	100	Pyrosequencing	^92^
*MINT25*	48	20	90	95.8	Pyrosequencing	^93^
*RORA*	48	19	60	85.4	Pyrosequencing	
*GDNF*	47	20	65	89.6	Pyrosequencing	
*ADAM23*	48	19	70	83.3	Pyrosequencing	
*PRDM5*	48	20	65	93.7	Pyrosequencing	
*MLF1*	48	20	60	85.4	Pyrosequencing	
*MINT25/PRDM5/ADAM23*	48	20	90	91.7	Pyrosequencing	
*MINT25/PRDM5/GDNF*	48	20	95	91.7	Pyrosequencing	

Note: MSP, methylation-specific PCR; MCTA-seq, methylated CpG tandem amplification and sequencing; q-PCR, quantitative real-time PCR; q-MSP, quantitative methylation-specific PCR; Hi-SA, high-sensitivity assay for bisulfite DNA.

### Leukocytes in peripheral blood

The peripheral blood can provide real-time information from tumors rich in leucocytes.^94^ Leukocytes are considered the first line of defense in the immune system against cancer, and they first cause changes in the epigenetic characteristics of peripheral leukocytes during immunoediting once tumorigenesis occurs.^95^ Therefore, changes in the epigenetic characteristics of leukocytes can directly reflect the immune response triggered by tumorigenesis and progression. Meanwhile, the membrane structure of leukocytes can ultimately preserve tumor-related biological information, which makes the substances carried by leukocytes have great potential as new tumor biomarkers. Previous studies of leukocyte genomic DNA (gDNA) methylation levels have identified several markers consistent with variants of precancerous gastric disease. For example, in a 5-year GC follow-up study, the hypermethylation status of *KIBRA*, *EPHA5*, *FAT4*, *DLEC1*, and *WT1*, and the hypomethylation status of *ZNF331* in the gDNA of leukocytes in the experimental group are considerably different compared with the control group, suggesting their association with gastric carcinogenesis.^96^, ^97^, ^98^, ^99^, ^100^, ^101^ Furthermore, Rusiecki et al have found the pattern of global hypomethylation and TP53 promoter hypermethylation of peripheral blood leukocytes in the case group compared with the control group.^102^ Beyond that, some studies have been concerned with the methylation patterns of REs (LINE-1 and Alu) in peripheral blood leukocytes from GC patients, while, no unified conclusion has been reached so far. Dauksa et al and Hou et al have confirmed that the average methylation levels of LINE-1 and Alu repeat sequences in leukocytes of GC patients are significantly lower, showing excellent application prospects in screening early GC.^103^^,^^104^ Nevertheless, Gao et al and Barchetta et al have found that Alu methylation in peripheral blood leukocyte DNA is negatively correlated with GC.^105^^,^^106^ These results suggest that although these autonomous transposable elements are good indicators of global DNA methylation status, more individual studies are required to confirm the clinical applicability of LINE-1 and Alu in leukocytes for GC.

### Cell-free DNA (cfDNA)

Circulating cfDNA is released into body fluids from apoptotic cells in the form of single- or double-stranded DNA. In cancer patients, the cfDNA released into the bloodstream by tumor cells hides tumor-specific variants of the original tumor, called circulating tumor DNA (ctDNA).^107^ During tumor cell turnover or other forms of cell death, DNA fragments from tumor cells are shed into peripheral blood leading to a several-fold increase in ctDNA levels in cancer patients than in healthy individuals. Moreover, the half-life of ctDNA is less than 2 h compared with the weeks-long half-life of traditional protein markers, which can more accurately reflect the real-time tumor burden.^108^ In 2020, the early cancer detection company GRAIL released the most comprehensive research results of the Circulating Cell-free Genome Atlas project so far. This study includes 6689 participants (2482 with cancer and 4207 with non-cancer) and provides convincing data that the liquid biopsy-based cfDNA methylation approach can detect all stages of over 50 cancer types, including GC. The sensitivity of cfDNA for detecting early-stage (I–III) GC is 78%, and the specificity is 99%, proving the feasibility of cfDNA methylation for GC early detection.^109^ Several pieces of research have shown that serum or plasma ctDNA methylation can be used as a biomarker to detect GC early. Balgkouranidou et al used MSP testing in blood samples from 73 GC patients and found that the methylated status of *SOX17* promoter could be examined in 43 plasma cfDNA but not in the serum of healthy people.^84^ Similarly, in another study, Lin et al have determined that the methylated rates of selected cfDNAs, *RUNX3*, *ZIC1*, and *HOXD10*, are increased during the process of gastric tumorigenesis, and the combination of these three genes is more statistically significant than a single biomarker in terms of predicting GC.^81^

Based on the distribution characteristics of CpG short tandem sequences in the human genome, Wen et al have developed a novel technique, methylated CpG tandems amplification and sequencing (MCTA-Seq), which can analyze cfDNA abnormally hypermethylated CGIs at the genomic scale with high sensitivity.^110^ Ren et al have further applied this MCTA-Seq technology to a noninvasive early screening of GC. By analyzing 89 plasma specimens from GC patients, 82 control plasma specimens, and 56 pairs of GC and adjacent tissues, cfDNAs including *DOCK10*, *CABIN1*, and *KCNQ5*, are identified to detect and discriminate GC, providing strong data support for noninvasive blood screening of GC.^74^ Some current studies have combined several DNA-methylated sites with conventional protein biomarkers in a panel to exhibit maximal detection capability. In a prospective study of 518 participants, Xu et al reported that the panel of methylated *Septin9* (*SEPT9*) and *RNF180* detected 60.3% of GC, while the sensitivity was increased to 68.6% when combined with CA72-4.^111^

To benefit GC patients in clinical practice, a few clinical trials are ongoing to confirm whether cfDNA methylation can be used as a biomarker for early screening of GC. An ongoing clinical trial (NCT04511559) characterizes cfDNA methylation in GC and evaluates its potential clinical application as a diagnostic and prognostic indicator of GC. Another larger sample size trial (NCT05336058) involving 1240 participants has screened poly-cfDNA specific methylation signatures in diagnosing GC of different types and stages. In addition, a prospective trial (NCT05224596) has enrolled 128 patients of benign gastric diseases and 370 GC patients to identify an early warning model based on cfDNA methylation. Data based on clinical trials will provide a basis for the subsequent development of methylation site-based cancer detection kits. *RNF180*/*SEPT9* Non-invasive Screening Test Kit has been certified by National Medical Products Administration (NMPA) in China and introduced to the GC early screening market. By measuring the methylation levels of *RNF180* and *SEPT9* in peripheral blood, this kit can preliminarily determine whether a person is likely to develop GC.

Moreover, feedback data from 23,000 tests show that compared with gastroscopy, the kit has the advantages of being readily accepted by patients and a one-time blood draw without interruption, which is of great value in improving the early screening rate of GC and reducing the cost of treatment. Similarly, Professor Bradley W. Anderson of Mayo Medical Center has developed a panel of the informative methylated DNA (*ARHGEF4*, *ABCB1*, *ELMO1*, *CLEC11A*, *SFMBT2*, *ST8SIA1*, *CD1D*, *ZNF569*, *C13ORF18*, and *CYP26C1*) using specimens collected from American and Korean patients for early detection of GC, which detects 100% GC in the United States and 94% in South Korea, with a specificity of 95%.^112^ However, it is necessary to perform additional exploration of these methylated DNA sites with larger sample sizes for the clinical promotion of this panel.

### Fecal DNA

Fecal DNA methylation biomarkers are an effective screening tool for gastrointestinal tumors. Its related product, *SDC2* methylation-based EarlyTect™-Colon Cancer test, has been approved by the U.S. Food and Drug Administration and the NMPA for colorectal cancer screening.^113^^,^^114^ Although no products are commercially available in the field of GC screening, several completed studies have confirmed the feasibility of fecal DNA methylation. For example, Goel's team, for the first time, in 2009, has put forward the potential of non-invasive biomarkers based on DNA methylation in the gastrointestinal tract and indicated that methylation of SFRP2 and RASSF2 gene promoters in feces is relevant to gastric and intestinal tumors, providing a noninvasive method for screening GC.^90^^,^^115^ In addition, Cao et al have developed a “ColoCaller” test to simultaneously detect the methylation status of *TFPI2*, *NDRG4*, *WIF1*, and *SDC2* in fecal DNA as a detected method for people at high risk of gastrointestinal tumors.^91^ In another cohort of fecal samples from 156 GC patients, Guo et al have shown that the combination of fecal occult blood testing composed of *SDC2* methylation and *TERT* methylation performs well in screening for GC, with the highest sensitivity in identifying gastric stage I cancer and gastric body cancer (78.6% and 75.0%, respectively).^89^ Liu et al have shown that fecal TERT promoter methylation analysis can be used for non-invasive gastrointestinal cancer screening.^88^ Besides, several existing studies are based on methylation multiple-site joint detection for GC screening as the specificity and sensitivity of single gene methylation for tumor diagnosis are of poor value.

### Gastric juice

Gastric juices are produced from the mucosal layer of the stomach where most stomach cancer starts.^116^ Therefore, cancer cells from the gastric mucosal layer can be directly released into gastric juice. In theory, gastric juice is an excellent noninvasive source of biomarkers for GC screening. In 2008, Muretto et al proposed that DNA collected from exfoliated cells in the gastric fluid can be used to analyze *CDH1* promoter hypermethylation. The results show that the *CDH1* promoter is widespread hypermethylation in the patient group.^117^ In practice, however, it is not feasible to use gastric juice DNA for molecular diagnosis because DNA is easily damaged by stomach acid (strongly acidic, pH less than 3).^118^^,^^119^ Watanabe et al have proposed an alternative to the highly acidic gastric juice for molecular analysis using gastric washes, which is the flushing fluid (gastric mucosa and normal saline) obtained during routine endoscopy. Of the six hypermethylated genes (*RORA*, *ADAM23*, *MLF1*, *MINT25*, *GDNF*, and *PRDM5*), *GDNF* and *MINT25* are the most sensitive biomarkers for early GC, and in terms of gastric washes, the MINT25 site has the best specificity (95.8%) and sensitivity (90%).^93^

Similarly, the lipid bilayers of exosomes can also stabilize the encapsulated DNA, preventing it from degradation and denaturation by extracellular gastric juice.^120^ By analyzing the gastric juice-derived exosomal DNA (exoDNA), Yamamoto et al have found that exo-*BARHL2* methylation can be detected in diffuse GC, indicating that methylated exo-*BARHL2* may be useful for early GC in clinical settings.^92^ Furthermore, exoDNA methylation is not affected by gastric mucosal atrophy or *H. pylori* infection, supporting that exosomes from gastric juice may be another alternative for molecular screening of GC.^92^

## Development of techniques for DNA-methylation analysis

**Figure 3:**
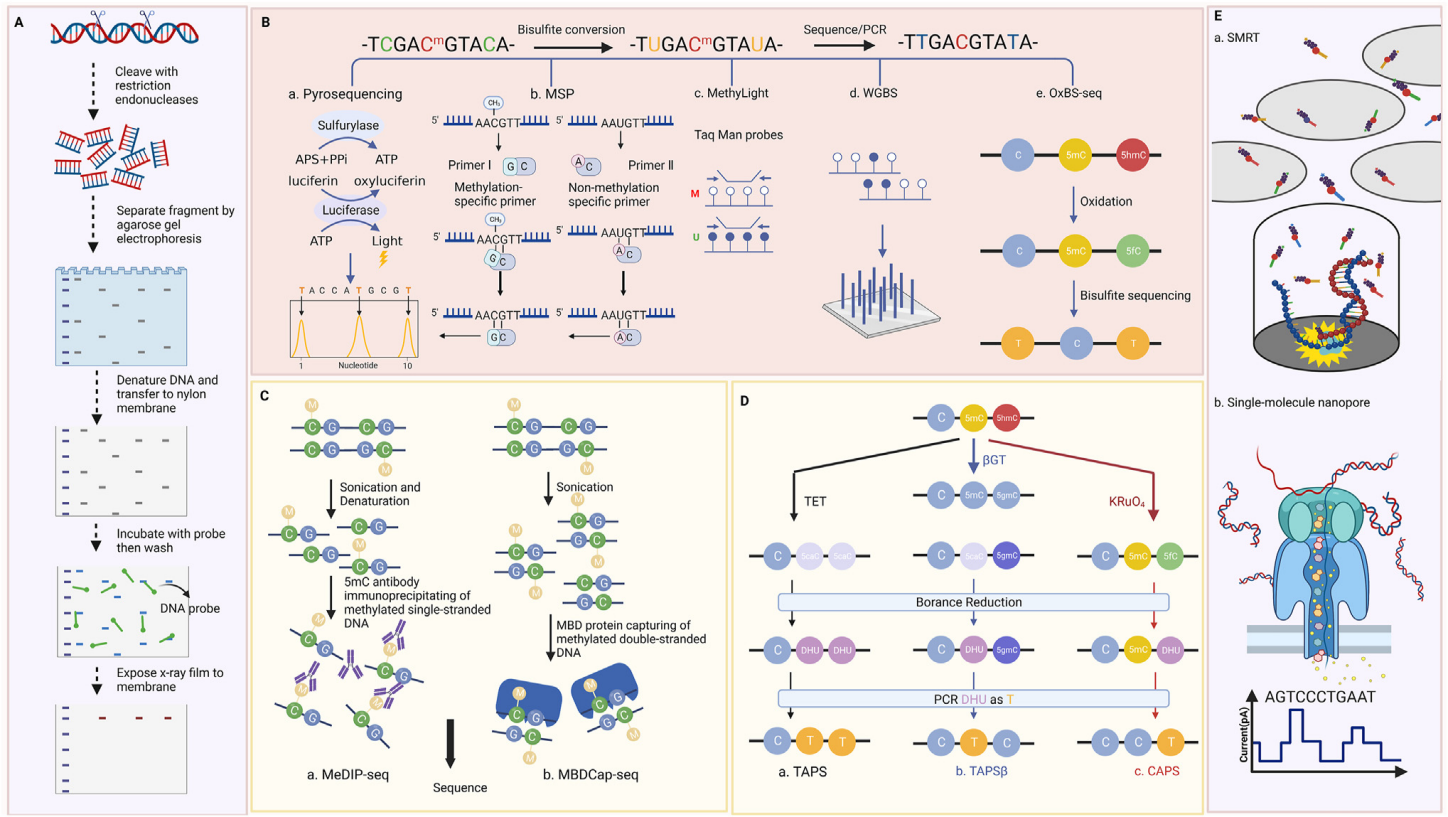
Detection techniques for DNA methylation. **(A)** Methods based on endonuclease digestion. **(B)** Techniques based on bisulfite treatment. a. Pyrosequencing; b. MSP; c. MethyLight; d. WGBS; e. oxBS-seq. **(C)** Technology based on affinity enrichment: a. MeDIP-seq; b. MBD-seq. **(D)** Bisulfite-independent DNA methylation sequencing: a. TAPS; b. TAPSβ; c. CAPS. **(E)** TGS: a. SMRT; b. single-molecule nanopore DNA sequencing. CAPS, chemical-assisted pyridine borane sequencing; MBD-seq, methyl-CpG binding domain protein-enriched genome sequencing; MeDIP-Seq, methylated DNA immunoprecipitation sequencing; MSP, methylation-specific PCR; WGBS, whole-genome bisulfite sequencing; oxBS-seq, oxidative bisulfite sequencing; SMRT, single-molecule real-time sequencing; TAPS, TET-assisted pyridine borane sequencing; TAPSβ, TET-assisted pyridine borane sequencing with β -glucosyltransferase; TGS, third-generation sequencing.

Exploring susceptible and reliable methylation detection methods is paramount for its clinical translation. Up to now, methylation detection technology has undergone a tremendous revolution. Briefly, the methods for detecting DNA methylation can be divided into the following categories: methods based on endonuclease digestion, methods based on bisulfite treatment, technology based on affinity enrichment, bisulfite-independent DNA methylation sequencing, and third-generation sequencing (Fig. 3).

### Methods based on endonuclease digestion

In restriction endonuclease pretreatment-based methylation detection methods, DNA methylation is the first treatment with restriction endonuclease HpaII, thereby protecting the methylated CpG sites, while unmethylated CpGs are enzymatically digested.^121^^,^^122^ In a later study, Waalwijk et al found that Msp I, a subtype of HpaII, cleaves DNA at the same site as HpaII, independent of their methylation status.^122^ However, the endonuclease treatment can only identify cytosines before the CpG site (CCGG) and thus cannot accurately display the complete profile of genome methylation.

### Methods based on bisulfite treatment

Bisulfite-sequencing (BS-Seq), developed by Frommer et al, is a milestone in the history of DNA methylation research.^123^ Under bisulfite treatment, non-methylated cytosine nucleotides are converted to uracil, which is interpreted as thymine when sequenced. Therefore, bisulfite produces a different base composition in methylated DNA than in unmethylated DNA, which can be easily quantified using conventional sequencing techniques. Methods based on bisulfite treatment for DNA methylation detection and analysis are still mainstream, and commonly used methods include pyrosequencing, MSP, methyLight, whole-genome bisulfite sequencing (WGBS), and oxidative bisulfite sequencing (oxBS-seq).

**Pyrosequencing**. As a real-time sequencing technology that can quantify DNA methylation at the single base resolution, pyrosequencing has been regarded as the gold standard of methylation quantitative analysis.^124^^,^^125^ Pyrosequencing was developed by Ronaghi et al in 1987, and its core is an enzymatic cascade reaction by DNA-polymerase, ATP-sulfurylase, luciferase, and apyrase in the same reaction system.^124^^,^^125^ The principle is that after the template DNA is annealed, under the synergistic action of the above four enzymes, each dNTP polymerization reaction is coupled and releases the fluorescence signal. The nucleotide sequence of the template DNA is recorded in real-time in the form of a fluorescent signal. This technology is especially suitable for sequencing analysis of known short sequences due to its advantages of sensitivity, rapidity, accuracy, reproducibility, and automation.^126^ In addition, pyrosequencing has been used for promoter methylation analysis in the early detection of GC, such as the *COX-2* gene promoter.^127^ However, the wide clinical practice of pyrosequencing has been limited due to its relatively high cost and cumbersome steps, although it is regarded as the gold standard.

**MSP**. Compared with pyrosequencing, MSP is more cost-effective and requires no special instruments, making it the most widely used method today. Wang et al developed this method by treating gDNA with bisulfite, whereby all unmethylated cytosine is converted to uracil while the methylated cytosines remain unchanged. Subsequently, two pairs of primers are designed. One pair of MSP primers is used to amplify the bisulfite-treated DNA template, while the other pair amplifies the unmethylated fragment.^128^ The detection site is methylated if the first primer pair can amplify the fragment. If the second primer pair can amplify the fragment, then the detection site is unmethylated. This method is susceptible and can be used for DNA methylation detection in paraffin-embedded samples, and endonucleases do not limit it. However, this approach highlights the critical need for specific and optimized primers designed for target regions to avoid spurious DNA amplification.

**MethyLight**. Firstly, the methyLight method treats the DNA fragment with bisulfite and designs a probe complementary to the site to be detected, followed by real-time quantitative PCR.^129^^,^^130^ The most significant advantage of this method is its high throughput and sensitivity. In addition, it eliminates the need for post-PCR operations such as electrophoresis and hybridization, reducing contamination and operational errors.^130^ Alarcón et al have applied the methyLight assay in 25 GC patients and 25 healthy individuals to assess the contribution of RPRM DNA methylation to the diagnosis of GC.^85^

**WGBS**. On a genome-wide scale, WGBS can accurately detect the methylation level of all individual cytosine groups. This method is currently widely used in methylation research due to the following reasons: (i) WGBS can be applied to all species with known reference genomes, including humans and most animals and plants; (ii) WGBS can obtain complete methylation information and accurate methylated mapping of the whole genome to the maximum extent; (iii) WGBS has high reliability and can directly sequence and quantitate methylated fragments without cross-reaction and background noise.^131^ Recent evidence has shown the broad application of WGBS. For example, Habibi et al comprehensively and accurately detected DNA methylation modifications in two types of mouse embryonic stem cells and performed a systematic comparison.^132^

**OxBS-seq.** In BS-seq, both 5 mC and 5 hmC are regarded as cytosines and, therefore, cannot be distinguished. In contrast, oxBS-seq employs chemical oxidation to convert oxidizing 5-hydroxymethylcytosine (5 hmC) to newly formed 5-formylcytosine (5 fC) at single-base resolution in gDNA. Then 5 fC and unmodified cytosine are converted to uracil, while 5 mC is not converted to cytosine.^132^ In this way, 5 hmC can be precisely discriminated from 5 mC by sequencing.

### Technology based on affinity enrichment

There are two main types of DNA methylation detection techniques based on affinity enrichment, methylated DNA immunoprecipitation (MeDIP) and methyl-CpG binding domain-based proteins (MBDCap). The former enriches CPG hypermethylated regions, while the latter does the opposite. MeDIP sequencing (MeDIP-Seq) is a genome-wide methylation detection technique based on antibody enrichment for sequencing. At the same time, MBD-seq is highly specific, sensitive, and suitable for identifying areas of differential methylation. Furthermore, compared with MeDIP-seq, MBD-seq does not require DNA denaturation and is a cost-saving method that can be used for methylation detection of large samples.^133^, ^134^, ^135^

### Bisulfite-independent DNA methylation sequencing

Bisulfite-independent DNA methylation sequencing is defined as a bisulfite-free genome-wide 5 mC single-base resolution sequencing technology through chemical labeling and enzymatic enrichment means.^136^ Liu et al have utilized the DNA demethylation function of ten-eleven translocation (TET) enzymes to establish TET-assisted pyridine-borane sequencing (TAPS), in which 5 hmC and 5 mC are both oxidized by TET to 5-carboxyl cytosines and then reduced to dihydrouracil (DHU) by pyridine-boranes.^137^ DHU is subsequently amplified and sequenced to thymidine. This method can locate and analyze 5 mC and 5 hmC in the genome but cannot separate 5 mC and 5 hmC. Because of this, the TAPS method needs to be improved to achieve a 5 mC localization analysis. In this context, Liu et al have developed TET-assisted pyridine borane sequencing with β -glucosyltransferase (TAPSβ) and chemical-assisted pyridine borane sequencing (CAPS).^136^^,^^137^ TAPSβ and CAPS methods have been demonstrated for genome-wide 5 mC and 5 hmC discrimination.^136^, ^137^, ^138^ Furthermore, the TAPS method is highly sensitive, and only 10 ng of DNA is required to analyze the methylation of free DNA in peripheral blood.^139^

### Third-generation sequencing

Third-generation sequencing adopts the synthesis-by-sequencing strategy to obtain sequence information by replicating the template strand, mainly including single-molecule real-time sequencing (SMRT) and single-molecule nanopore DNA sequencing. Xiao et al have employed SMRT to study human DNA N^6^-methyladenine (m^6^A), identified 881,240 m^6^A modification sites in the human genome, and obtained the Chinese DNA m^6^A modification map for the first time.^140^ The idea of sequencing single-stranded RNA or DNA molecules using nanopores in membranes originated in the late 1980s.^141^ The biggest breakthrough in nanopore sequencing technology is the ability to sequence single-stranded DNA directly after the DNA has been unstranded. However, the disadvantages of nanopore technology are the high error rate (∼15%) and the inability to sequence the same strand multiple times as SMRT sequencing.^142^

## Conclusions and perspectives

Recent studies have found that abnormal DNA methylation is a hallmark of the precancerous or early stage of GC and has shown promise as a routine biomarker tool in clinical practice, mainly due to its accuracy, ease of collection, and minimal invasiveness. Understanding the factors that control DNA methylation status during gastric carcinogenesis and progression is of great biological and clinical importance in exploring the pathogenesis of GC and detecting biomarkers for early GC. Therefore, in the present review, we started with the links between DNA methylation and pathogenic genes of GC, focusing on the evidence that pathogens (*H. pylori* and EBV) and aging are associated with DNA methylation in GC. Then we provided a list-based overview of novel DNA methylation biomarker candidates for early detection of GC based on sample sources, including leukocytes in peripheral blood, plasma, feces, gastric juice, and gastric washes. Finally, translating GC DNA methylation biomarkers from the laboratory to the clinic depends on technological breakthroughs in highly sensitive and reliable methylation detection methods. Here, we provided a practical summary of DNA methylation analysis techniques.

Although there is a better prospect of studying DNA methylation as a biomarker for GC, there are still some unresolved issues. Even though many studies have identified GC-specific DNA methylation characteristics, most of these have been observed in a single study. Therefore, we attempted to summarize the use of DNA methylation in GC. However, due to confusion caused by inconsistent experimental conclusions, more studies are needed to understand the molecular processes involved in DNA methylation and gastric carcinogenesis. Moreover, the road of DNA methylation biomarkers from laboratory to clinical translation is long and costly. To date, over 100 DNA methylation-based biomarker candidates have been proposed with great clinical promise in the early detection of GC. However, to our knowledge, only a few candidates have been applicated clinically in the past decade. The entire process of biomarker clinical translation involves biomarker identification, testing, validation, and then clinical evaluation, followed by manufacturing, use development, and certification. In addition, new biomarkers need to perform better, be less invasive, and be more cost-efficient than existing clinical trials and clinical methods, which are the prerequisites to obtain the required investment for their translation to the clinic for early GC screening. The methylome-level data obtained from the studies described in our review will certainly spur future independent studies dedicated to finding candidate DNA methylation biomarkers, which could provide a complete source of DNA methylated changes during the early progression of GC.

## Author contributions

CXW and LTD conceived the project. HZ, YQD, TSM, and GM collected the literature and drafted the manuscript. XHW and YHZ did the literature review, drew the figures, and drafted the manuscript. CXW and LTD revised the manuscript. All authors read the final manuscript and agreed to its publication.

## Conflict of interests

The authors declare no conflict of interests.

## Funding

This work was supported by the 10.13039/501100001809National Natural Science Foundation of China (No. 82202611, 82202633), 10.13039/501100002858China Postdoctoral Science Foundation (No. 2022M711912, BX20220194), 10.13039/501100007129Natural Science Foundation of Shandong Province, China (No. ZR2022QH031), Natural Science Foundation of Jiangsu Province, China (No. BK20220271), and Fundamental Research Funds of the Second Hospital of 10.13039/100009108Shandong University, Shandong, China (No. 2022YP01).
